# Proposed declassification of disease categories related to sexual orientation in the *International Statistical Classification of Diseases and Related Health Problems* (ICD-11)

**DOI:** 10.2471/BLT.14.135541

**Published:** 2014-06-17

**Authors:** Susan D Cochran, Jack Drescher, Eszter Kismödi, Alain Giami, Claudia García-Moreno, Elham Atalla, Adele Marais, Elisabeth Meloni Vieira, Geoffrey M Reed

**Affiliations:** a Department of Epidemiology, Fielding School of Public Health, 640 Charles E Young Dr S, University of California, Los Angeles, California, 90024-1772, United States of America (USA).; b New York Medical College, New York, USA.; c Consultant, World Health Organization, Geneva, Switzerland.; d Centre for Research in Epidemiology and Population Health, Institut de la Santé et de la Recherche Médicale **(**INSERM), Kremlin-Bicêtre, France.; e Department of Reproductive Health and Research, World Health Organization, Geneva, Switzerland.; f Primary Care and Public Health Directorate, Ministry of Health, Manama, Bahrain.; g Department of Psychiatry and Mental Health, University of Cape Town, Cape Town, South Africa.; h Ribeirão Preto Medical School, University of São Paulo, São Paulo, Brazil.; i Department of Mental Health and Substance Abuse, World Health Organization, Geneva, Switzerland.

## Abstract

The World Health Organization is developing the 11th revision of the *International Statistical Classification of Diseases and Related Health Problems* (ICD-11), planned for publication in 2017. The Working Group on the Classification of Sexual Disorders and Sexual Health was charged with reviewing and making recommendations on disease categories related to sexuality in the chapter on mental and behavioural disorders in the 10th revision (ICD-10), published in 1990. This chapter includes categories for diagnoses based primarily on sexual orientation even though ICD-10 states that sexual orientation alone is not a disorder. This article reviews the scientific evidence and clinical rationale for continuing to include these categories in the ICD. A review of the evidence published since 1990 found little scientific interest in these categories. In addition, the Working Group found no evidence that they are clinically useful: they neither contribute to health service delivery or treatment selection nor provide essential information for public health surveillance. Moreover, use of these categories may create unnecessary harm by delaying accurate diagnosis and treatment. The Working Group recommends that these categories be deleted entirely from ICD-11. Health concerns related to sexual orientation can be better addressed using other ICD categories.

## Résumé

L'Organisation mondiale de la Santé est en train de mettre au point la 11e révision de la *Classification statistique internationale des maladies et des problèmes de santé connexes* (CIM-11), dont la publication est prévue pour 2017. Le Groupe de travail sur la Classification des troubles sexuels et de la santé sexuelle a été chargé d'examiner et de faire des recommandations sur les catégories de maladies liées à la sexualité dans le chapitre sur les troubles mentaux et comportementaux de la 10e révision (CIM-10) qui a été publiée en 1990. Ce chapitre comprend les catégories des diagnostics basés principalement sur l'orientation sexuelle même si la CIM-10 stipule que l'orientation sexuelle seule n'est pas un trouble. Cet article examine les données scientifiques et les raisons cliniques pour continuer à inclure ces catégories dans la CIM. Un examen des données publiées depuis 1990 a révélé le peu d'intérêt scientifique pour ces catégories. En outre, le Groupe de travail n'a trouvé aucune preuve de leur utilité clinique: elles ne contribuent pas à la fourniture des services de soins ou à la sélection du traitement, et elles ne fournissent aucune information essentielle en matière de surveillance de la santé publique. Par ailleurs, l'utilisation de ces catégories peut créer des dommages inutiles en retardant le diagnostic précis et le traitement. Le Groupe de travail recommande que ces catégories soient entièrement supprimées de la CIM-11. Les problèmes de santé liés à l'orientation sexuelle peuvent être mieux traités en utilisant les autres catégories de la CIM.

## Resumen

La Organización Mundial de la Salud está desarrollando la undécima revisión de la *Clasificación Estadística Internacional de Enfermedades y Problemas Relacionados con la Salud* (CIE-11), cuya publicación está planeada para el 2017. El Grupo de Trabajo sobre la Clasificación de Trastornos Sexuales y Salud Sexual fue encargado de revisar y hacer recomendaciones sobre estas categorías de enfermedades relacionadas con la sexualidad en el capítulo sobre trastornos mentales y del comportamiento en la décima revisión (CIE-10), publicada en 1990. Este capítulo incluye categorías para diagnósticos basadas principalmente en la orientación sexual, a pesar de que la CIE-10 afirma que la orientación sexual en sí misma no es un trastorno. Este artículo revisa las pruebas científicas y los fundamentos clínicos para continuar incluyendo estas categorías en la CIE. Una revisión de las pruebas publicada desde 1990 encontró poco interés científico en estas categorías. Asimismo, el Grupo de Trabajo no encontró pruebas de que fueran útiles clínicamente: no contribuyen a la prestación de servicios sanitarios ni a la selección de tratamientos. Tampoco proporcionan información esencial para la vigilancia de la salud pública. Además, el empleo de estas categorías podría ocasionar un daño innecesario al retrasar los diagnósticos precisos y el tratamiento. El Grupo de Trabajo recomienda que se eliminen totalmente estas categorías de la CIE-11. Los problemas de salud relacionados con la orientación sexual se pueden abordar mejor utilizando otras categorías de la CIE.

## ملخص

تقوم منظمة الصحة العالمية بوضع المراجعة الحادية عشرة للتصنيف الإحصائي الدولي للأمراض والمشكلات المتعلقة بالصحة (ICD-11)، المخطط نشرها عام 2017. وتم تكليف الفريق العامل المعني بتصنيف الاضطرابات الجنسية وجوانب الصحة الجنسية بمسؤولية مراجعة وإصدار التوصيات بشأن فئات الأمراض ذات الصلة بالميول الجنسية في الفصل المعني بالاضطرابات النفسية والسلوكية في المراجعة العاشرة (ICD-10)، التي تم نشرها عام 1990. ويشمل هذا الفصل فئات التشخيصات المستندة بشكل أساسي على التوجه الجنسي رغم إقرار المراجعة العاشرة للتصنيف الإحصائي الدولي للأمراض والمشكلات المتعلقة بالصحة بأن التوجه الجنسي وحده ليس اضطراباً. وتستعرض هذه المقالة البيّنات العلمية والأساس المنطقي السريري للاستمرار في إدراج هذه الفئات في التصنيف الإحصائي الدولي للأمراض. وتوصلت مراجعة البيّنات التي يتم نشرها منذ عام 1990 إلى وجود قدر ضئيل من الاهتمام العلمي بهذه الفئات. بالإضافة إلى ذلك، لم يجد الفريق العامل أي بيّنات تشير إلى أنها مفيدة سريرياً: لا تسهم في إيتاء الخدمات الصحية أو اختيار العلاج ولا توفر معلومات أساسية لترصد الصحة العمومية. علاوة على ذلك، من الممكن أن يتسبب استخدام هذه الفئات في أضرار غير لازمة عن طريق تأجيل التشخيص الدقيق والعلاج. ويوصي الفريق العامل بحذف هذه الفئات بالكامل من المراجعة الحادية عشرة للتصنيف الإحصائي الدولي للأمراض والمشكلات المتعلقة بالصحة. ويمكن التعامل مع مخاوف الصحة ذات الصلة بالتوجه الجنسي بشكل أفضل باستخدام فئات التصنيف الإحصائي الدولي للأمراض.

## 摘要

世界卫生组织正在进行《疾病和有关健康问题的国际统计分类》(ICD-11)的第11次修订,修订版计划于2017年公布。性疾病和性卫生分类工作组负责审查1990年公布的第10次修订(ICD-10)中有关精神和行为障碍章节中与性相关的疾病分类,并提出建议。该章包括主要基于性取向的诊断类别,尽管ICD-10指出性取向本身并不是一种疾病。本文综述了继续在ICD包含这些类别的科学证据和临床理由。针对1990年以来的证据发表一篇综述几乎找不到这些类别有什么科学意义。此外,工作组也没有找到证据表明这些类别在临床上有用:既无益于健康服务交付或治疗选择,对公共卫生监测也没有提供重要信息。此外,使用这些类别可能因为推迟准确的诊断和治疗而产生不必要的伤害。工作组建议在ICD-11中完全删除这些类别。与性取向相关的健康问题可以使用其他ICD类别更好地解决。

## Резюме

Всемирная организация здравоохранения разрабатывает 11-ю редакцию *Международной статистической классификации болезней и проблем, связанных со здоровьем* (МКБ-11), которую планируется опубликовать в 2017 году. Рабочей группе по классификации сексуальных расстройств и сексуальному здоровью было поручено рассмотреть и разработать рекомендации по категориям заболеваний, связанных с сексуальностью, в главе о психических и поведенческих расстройствах в 10-й редакции (МКБ-10), опубликованной в 1990 году. В этой главе содержатся категории диагнозов, основанных в первую очередь на сексуальной ориентации, хотя в МКБ-10 указывается, что сексуальная ориентация сама по себе не является расстройством. В этой статье рассматриваются научные доказательства и клиническое обоснование сохранения этих категорий в МКБ. Обзор данных, опубликованных с 1990 года, свидетельствует о низкой научной заинтересованности в сохранении этих категорий. Кроме того, рабочая группа не обнаружила никаких доказательств их клинической полезности: они не способствуют оказанию медицинских услуг или выбору методов лечения и не предоставляют необходимую информацию по улучшению наблюдения за состоянием общественного здравоохранения. Кроме того, использование этих категорий может причинить ненужный вред из-за задержки постановки точного диагноза и назначения лечения. Рабочая группа рекомендует полностью исключить эти категории из МКБ-11. Проблемы со здоровьем, связанные с сексуальной ориентацией, могут лучше решаться с помощью других категорий МКБ.

## Introduction

A core constitutional responsibility of the World Health Organization (WHO) is the development and maintenance of international health classification systems such as the *International Statistical Classification of Diseases and Related Health Problems* (ICD)^1^ (Box 1). Currently, WHO is revising the ICD and it is anticipated that the 11th revision (ICD-11) will be published in 2017. As part of this process, WHO’s Departments of Mental Health and Substance Abuse and Reproductive Health and Research have appointed a Working Group on the Classification of Sexual Disorders and Sexual Health (hereafter referred to as the Working Group). The group is charged with reviewing and making recommendations pertaining to categories related to sexuality in the chapter on mental and behavioural disorders in the previous version; ICD-10. Before making its recommendations, the Working Group was asked to consider the substantial scientific advances that have taken place since 1990, when ICD-10 was published.

Box 1 **Structure of the *International Statistical Classification of Diseases and Related Health Problems, 10th revision***

*The International Classification of Diseases and Related Health Problems* (ICD) is the official classification of diseases, health conditions and related health problems of the World Health Organization (WHO). It is used to assign human morbidity and mortality to specific categories.

The 194 Member States of WHO agree to use the ICD as the standard for collecting and reporting information related to health conditions. This allows for the systematic tracking of mortality, morbidity and disease burden internationally and throughout time.

The ICD is also used to direct clinical care and research, allocate resources and monitor progress in achieving public health goals.

The classification is organized into 21 chapters, each containing disease or health-related categories or both, including:

**Chapter V. Mental & behavioural disorders**

F categories include the F-66 categories: psychological and behavioural disorders associated with sexual development and orientation

**Chapter XXI. Factors influencing health status and contact with health services**

Z categories include the Z-70 categories: counselling related to sexual attitude, behaviour and orientation

In ICD-10, mental and behavioural disorders include “Psychological and behavioural disorders associated with sexual development and orientation” coded as the F66 categories (Table 1). Although F66 categories mention gender identity, historically the categories emerged from earlier classifications of sexual orientation. The Working Group recommends that the F66 categories should be deleted in their entirety. In this paper, the authors, who participated in the Working Group, summarize the rationale for this recommendation, with particular reference to concerns about sexual orientation. A review of the Working Group’s recommendations on gender identity has been published elsewhere.^2^

**Table 1:** F66 categories in ICD-10: psychological and behavioural disorders associated with sexual development and orientation^a^

Code^b^	Category name	Description
F66.0	Sexual maturation disorder	The individual is uncertain about his or her gender identity or sexual orientation, which causes anxiety or depression. Most commonly this occurs in adolescents who are not certain whether they are homosexual, heterosexual or bisexual in orientation and in individuals who, after a period of apparently stable sexual orientation and often within a long-standing relationship, find that their sexual orientation is changing
F66.1	Ego-dystonic sexual orientation	The gender identity or sexual preference is not in doubt, but the individual wishes it were different because of associated psychological and behavioural disorders, and may seek treatment to change it
F66.2	Sexual relationship disorder	The gender identity or sexual preference abnormality is responsible for difficulties in forming or maintaining a relationship with a sexual partner
F66.8	Other psychosexual development disorders	NA
F66.9	Psychosexual development disorder, unspecified	NA

ICD-10: *International Statistical Classification of Diseases and Related Health Problems*, 10th revision; NA: not applicable.

^a^ Sexual orientation alone is not to be regarded as a disorder.

^b^ The following five-character codes may be used to indicate variations of sexual development and orientation that may be problematic for the individual: F66.x0 – heterosexual; F66.x1 – homosexual; F66.x2 – bisexual (to be used only when there is clear evidence of sexual attraction to members of both sexes); and F66.x8 – other, including prepubertal, where x is the figure after the decimal point for the relevant category in the table.

Sexual orientation is a contentious topic: internationally, homosexuality and other forms of expression of same-sex orientation are stigmatized.^3^^,^^4^ In 1948, WHO published ICD-6, which was the first ICD version to include a classification of mental disorders. Although ICD-6 classified homosexuality as a sexual deviation that was presumed to reflect an underlying personality disorder, subsequent research did not support this view.^5^ Moreover, recent surveys demonstrate that homosexual behaviour is a widely prevalent aspect of human sexuality.^6^^–^^15^ Over the last half century, several classification systems,^16^^,^^17^ including the ICD,^1^ have gradually removed diagnoses that once defined homosexuality per se as a mental disorder. These changes reflect both emerging human rights standards and the lack of empirical evidence supporting the pathologization and medicalization of variations in sexual orientation expression.^3^^,^^4^

It is explicitly stated in ICD-10 that “sexual orientation by itself is not to be considered a disorder”. Nevertheless, the descriptions of the F66 categories (Table 1) suggest that mental disorders exist that are uniquely linked to sexual orientation and gender expression. Our review of the merit of retaining these categories is guided by three basic principles: (i) optimizing clinical utility (e.g. identifying individuals who need mental health treatment and the services they require); (ii) meeting the needs of public health surveillance; and (iii) facilitating research.^18^ The review is also shaped by a fourth principle: awareness of human rights standards endorsed by the United Nations.^19^ As stated by the United Nations High Commissioner for Human Rights, “All people, including lesbian, gay, bisexual and transgender (LGBT) people, are entitled to enjoy the protections provided for by international human rights law, including in respect of rights to life, security of person and privacy, the right to be free from torture, arbitrary arrest and detention, the right to be free from discrimination and the right to freedom of expression, association and peaceful assembly”.^20^ International professional organizations, such as the World Association for Sexual Health and the International Planned Parenthood Federation, have also asserted that sexual rights, including rights pertaining to sexual orientation expression, are integral to human rights.^21^^,^^22^

## General considerations

Here, we consider several issues raised by the presence of the F66 categories in ICD-10 and how these issues have influenced the recommendations made by the Working Group.

### Mental disorder

An overriding issue is whether the F66 categories capture unique mental disorders, which raises the core question: What is a mental disorder? In 2011, the International Advisory Group for the Revision of ICD-10 Mental and Behavioural Disorders proposed retaining the following definition of mental and behavioural disorders from ICD-10: “a clinically recognizable set of symptoms or behaviours associated in most cases with distress and with interference with personal functions”.^23^ This definition is broad enough to encompass the great variety of mental disorders seen in clinical practice. However, it may be so broad that it could also include clinically recognizable syndromes such as grief responses to bereavement or reactions to everyday problems – syndromes that were not intended to be viewed as mental disorders.^24^ Consequently, the structure of the ICD categories distinguishes between mental disorders and psychological and emotional responses to particular life circumstances that may occur with or without a disorder. If a disorder is present, an appropriate diagnosis (i.e. a disorder category) may be applied. However, if no co-occurring disorder exists, a category from ICD-10’s chapter entitled “Factors influencing health status and encounters with health services” (i.e. the Z categories) may be used to indicate that an individual is seeking health services, including mental health services, in the absence of a current disorder. Existing Z categories include counselling related to sexual concerns (Box 1).

In addition, ICD-10 also recognizes that factors other than mental disorders may lead to behaviours or presenting complaints that could be misinterpreted as symptoms of disorders. Thus, the ICD states explicitly, “Social deviance or conflict alone, without personal dysfunction, should not be included in mental disorders.”^1^ This exclusion is essential because a variety of factors, including social environmental stressors and cultural norms,^25^ may lead to psychological experiences and behaviours that do not necessarily reflect an underlying disorder. In addition, social or political disapproval has at times resulted in the abuse of diagnoses – especially psychiatric diagnoses – to harass, silence or imprison people whose behaviour violates social norms or challenges existing authority structures.^26^

### Sexual orientation

Sexual orientation refers to a persistent tendency to experience sexual attractions, fantasies and desires and to engage in sexual behaviours with partners of a preferred sex. When individuals categorize themselves on the basis of their own sexual attractions, desires and behaviours, they are described as adopting a sexual orientation identity: for example, gay, lesbian or heterosexual. The causes of sexual orientation are unknown but are likely to reflect some mixture of genetics, prenatal hormonal exposure, life experience and social contextual factors.^27^

Four important conclusions can be drawn from surveys of sexual behaviour in several countries.^6^^–^^15^ First, variation in sexual orientation is ubiquitous, with the great majority identifying as heterosexual and a significant minority reporting other identities. Second, patterns of reported sexual identity and behaviour vary with sociodemographic characteristics, such as sex, age and race or ethnicity. For example, men are more likely to identify as gay rather than bisexual, whereas the reverse is the case for women. Third, there is evidence that inconsistent sexual orientation expression is associated with social and economic factors rather than psychopathology. Fourth, sexual orientation identity is not fixed for everyone and changes that occur throughout life do not always follow a linear pathway in or out of heterosexuality or homosexuality.^28^ Research on the development of sexual orientation expression, whether implicitly in general studies of adolescents and young adults^29^ or explicitly in studies focusing on lesbian, gay, bisexual and transgender individuals,^30^ has found that the onset of sexual behaviour, attraction and desire typically occurs in adolescence. These studies also found substantial variability in patterns of sexual expression both between individuals and within individuals across time. The patterns observed in adolescents differ from those observed in adults and are consistent with the gradual acquisition of experience with sexuality and the formation of close relationships. Among individuals with same-sex behaviour, attractions, or identity, a variable pattern is the norm rather than the exception. Given this variability, it is difficult to identify a distinct pattern of abnormal sexual orientation expression. Further, variation alone is an insufficient criterion for diagnosing a mental disorder.^1^^,^^23^

### Social deviance 

There is strong evidence that sexual orientation can be associated with substantial social stress.^31^ Same-sex orientation is linked to violence, stigma, exclusion and discrimination around the world.^32^ Violence against people perceived to be lesbian, gay, bisexual or transgender has been documented as especially vicious and often involves a high degree of brutality.^33^ International, regional and many national human rights bodies prohibit discrimination on the basis of same-sex orientation and have explicitly called on states to make all possible efforts to eliminate discrimination and prejudice.^34^ Further, several countries have legal provisions (e.g. hate crime statutes) that specifically address crimes committed on the basis of sexual orientation or gender identity.^35^ Nevertheless, in many countries, criminal law is still applied to consensual, same-sex, sexual activity.^32^ International, regional and national human rights bodies have explicitly called for states to end this practice.^36^^–^^39^

Consequently, the clause on the exclusion of social deviance in the ICD is particularly relevant in reviewing the F66 categories. If a disease label is to be attached to a social condition, it is essential that it has a demonstrable clinical utility, for example, by identifying a legitimate mental health need, and its use should not exacerbate existing stigma, violence and discrimination.

## Current F66 categories reviewed

### Sexual maturation disorder (F66.0)

The concept of psychosexual development, which has roots in psychoanalytical theories,^3^ refers to the development of one’s sense of gender identity, sexual orientation and gender role behaviours.^27^ According to Freudian theory, children are born with a diffuse set of sexual attractions that coalesce with age into a coherent heterosexual pattern of sexual expression. Presumed disruption of this hypothesized process is the conceptual basis for sexual maturation disorder. The core diagnostic features are: (i) uncertainty about one’s gender identity or sexual orientation; and (ii) distress about the uncertainty rather than about the particular gender identity or sexual orientation.

An immediate concern is whether sexual maturation disorder conflates developmental patterns within the normal range with pathological processes. Research repeatedly demonstrates that indicators of emerging same-sex sexual orientation are time-varying in their appearance, with the process beginning typically in late childhood or early adolescence.^30^ Further, during this time, people who exhibit a same-sex sexual orientation or gender nonconformity may also experience social stress arising from the stigma associated with same-sex orientations.^20^ However, such distress is not attributed to sexual maturation disorder because of the ICD’s social deviance exclusion.

### Ego-dystonic sexual orientation (F66.1)

The concept of ego-dystonic homosexuality was initially incorporated into mental disorders classifications as a part of the consensus-building process connected with the removal of homosexuality per se from the American Psychiatric Association’s *Diagnostic and Statistical Manual of Mental Disorders* in 1974.^40^ Homosexuality could still provide the basis for a diagnosis according to the manual but only if the individual was distressed about unwanted homosexuality. In 1987, even this diagnosis was removed. However, the concept was incorporated into ICD-10, which was approved in 1990, as part of a set of changes parallel to those made in the *Diagnostic and Statistical Manual of Mental Disorders* more than a decade before. Although homosexuality per se was removed as a diagnostic category in ICD-10, the classification describes ego-dystonic sexual orientation as follows: “the gender identity or sexual preference is not in doubt but the individual wishes it were different because of associated psychological and behavioural disorders and may seek treatment to change it”.^1^ The description invokes gender identity, but the intent, at least historically, was to address a clinical situation in which individuals express a desire to develop heterosexual attractions they do not feel or to relieve distress about an unwanted homosexual orientation.

Evidence shows that lesbian, gay and bisexual individuals often report a higher level of distress than heterosexuals. However, the elevated distress has been linked robustly to greater experiences of social rejection and discrimination.^41^ In the absence of an active desire to rid oneself of one’s current sexual orientation, distress related to sexual orientation does not fulfil the definition of ego-dystonic sexual orientation. Further, if distress results from social adversity, it falls under the ICD’s social deviance exclusion. There are several socially stigmatized conditions, such as physical illness or poverty,^42^^,^^43^ that are also likely to lead to distress. These conditions could be labelled “ego-dystonic” to the extent that they are unwanted but the ICD does not treat such distress as constituting a mental disorder.

### Sexual relationship disorder (F66.2)

Sexual relationship disorder describes a clinical syndrome in which an abnormal sexual preference or gender identity makes it difficult to form or maintain a relationship with a sexual partner. Generally ICD diagnoses reflect individual-level disturbances but the disturbance in sexual relationship disorder is dyadic by definition. Difficulties in relationships with sexual partners are commonplace and occur for many reasons. Moreover, ICD-10 does not include a classification for relationship disorders due to other potentially contributory factors. There is no justification for creating a mental disorder category that is specifically based on the co-occurrence of relationship problems with sexual orientation or gender identity issues.

### Other (F66.8), unspecified (F66.9) disorders

The category “Other psychosexual development disorders” is an exclusionary category that is used to classify disorders that clinicians determine to be psychosexual in nature but do not meet the requirements of the other F66 categories. Though ICD-10 does not define what constitutes a psychosexual developmental disorder, sexual orientation is clearly central to the concept given its prominence in the F66 categories. A major concern is that this category gives no specific information about what is being treated, nor does it indicate what might be appropriate treatment. Rather, it appears to provide an opportunity to apply an undefined mental disorder diagnosis to individuals with a same-sex orientation. This opportunity is also extended by the category “Psychosexual developmental disorder, unspecified”.

## Should F66 categories be retained?

Here, we consider whether or not the F66 categories should be retained in the context of the four basic principles that shaped the Working Group’s recommendations. In particular, we comment on their clinical utility, their use for public health surveillance and the negative consequences of their retention.

### Clinical utility

Clinical utility is enhanced when diagnostic categories provide useful information, are commonly understood by health-care providers and help select appropriate and effective interventions.^18^ In this context, one can ask: How are lesbian, gay and bisexual people currently treated in mental health care settings? **S**urveys of mental health practitioners in the United Kingdom of Great Britain and Northern Ireland and the United States of America reveal that the great majority have experience treating individuals with a same-sex orientation.^44^^,^^45^ Further, these individuals often seek services at an equal or higher rate than heterosexual individuals.^46^ These facts suggest that, if the F66 categories were actually in common use, there should be evidence of that use. Instead, however, it appears that people with a same-sex orientation typically receive treatment for common mental disorders, such as depression, anxiety disorders and problems with substance use. In the lone study of the content of common worries among people with a same-sex orientation, concerns about sexual orientation were relatively uncommon.^47^

One argument for retaining the F66 categories is that they may improve diagnostic accuracy because they can be used for individuals who present with concerns about sexual orientation or gender identity. For example, some practitioners may see sexual relationship disorder or sexual maturation disorder as an alternative diagnosis to a gender identity disorder. Similarly, distress about one’s life in the context of same-sex orientation may appear to warrant a diagnosis of ego-dystonic sexual orientation. However, it is not clear that assigning additional or alternative categories based on sexual orientation actually improves diagnostic accuracy, particularly given the problems with validity described above. On the contrary, the existence of these categories may be harmful because they draw attention to content (e.g. to a relationship breakup with a same-sex as opposed to different-sex partner) or individual characteristics that are not clinically meaningful or that pathologize normative reactions. In a search of Medline, Web of Science and PsycINFO databases, the Working Group found that the categories of sexual maturation disorder and sexual relationship disorder had generated no scientific publications as of 10 January 2014 publications. The last peer-reviewed reference to “ego-dystonic homosexuality” was published in 1995.^48^ Publications on psychosexual development do exist but we could not find any on psychosexual developmental disorders.

In addition, a literature search revealed no references to evidence-based treatment for F66 disorders. Moreover, there was no evidence that concern about gender identity or sexual orientation requires unique interventions that are substantially different from the common methods of treating distress, anxiety, depression and other mental disorders. The best clinical care for people with a same-sex orientation does not differ from that for their heterosexual counterparts.^49^ Therapies aimed at changing a person’s sexual orientation have been deemed outside the scope of ethical practice.^49^^,^^50^

On occasion, the argument is raised that the F66 categories might offer protection for people with a same-sex orientation in some countries. Currently, the Working Group is aware of six countries where same-sex sexual behaviour may be punishable by death. It has been argued that classifying some forms of same-sex sexual behaviour as mental disorders can protect individuals from execution for homosexuality via a mental disorder exemption. However, the Working Group was unable to establish whether such a defence has actually been used, despite sporadic executions for homosexuality in recent years. Further, retaining the F66 categories for this purpose alone is both inconsistent with human rights principles and the governing purpose of the ICD.

### Public health surveillance

An important role of the ICD is that it provides a common means of public health surveillance internationally. However, the F66 categories contribute little, if anything, to surveillance. They have not generated a body of research, are not routinely reported to WHO by any Member State and are not used in WHO’s calculations of the global burden of disease.

### Negative consequences

Retaining the F66 categories may create unnecessary harm. Individuals with a same-sex orientation may receive suboptimal care because use of these categories may lead to mistakes or delays in accurate diagnosis and treatment. Retention of these categories may also be construed as supporting ineffective and unethical treatment that aims to encourage people with a same-sex orientation to adopt a heterosexual orientation or heterosexual behaviour.^49^ From a human rights perspective, the F66 categories selectively target individuals with gender nonconformity or a same-sex orientation without apparent justification.^20^

## Recommendations

The Working Group recommends that the F66 grouping of categories entitled “Psychological and behavioural disorders associated with sexual development and orientation” be deleted in its entirety from ICD-11. Both concerns about gender identity and sexual orientation difficulties can well be addressed using other ICD categories. First, people with a same-sex orientation or gender nonconformity or who present with related concerns and who also meet the definitional requirements of a disorder (other than those covered by the F66 categories) can be diagnosed using existing categories. It is not justifiable from a clinical, public health or research perspective for a diagnostic classification to be based on sexual orientation. Second, the needs of individuals without a mental health or behavioural disorder can be classified using the Z categories if, for example, they require counselling related to sexuality. In this way, ICD-11 can address the needs of people with a same-sex orientation in a manner consistent with good clinical practice, existing human rights principles and the mission of WHO.
